# Acupressure in the control of migraine-associated nausea

**DOI:** 10.1007/s10072-012-1069-y

**Published:** 2012-05-30

**Authors:** Gianni Allais, Sara Rolando, Ilaria Castagnoli Gabellari, Chiara Burzio, Gisella Airola, Paola Borgogno, Paola Schiapparelli, Rita Allais, Chiara Benedetto

**Affiliations:** 1 Department of Gynecology and Obstetrics, Women’s Headache Center, University of Turin, Via Ventimiglia 3, 10126 Turin, Italy; 2 Department of Statistics and Applied Mathematics “Diego de Castro”, University of Turin, Turin, Italy

**Keywords:** Migraine, Nausea, PC6 acupressure

## Abstract

Migraine is a disabling neurological disorder, aggravated by accompanying symptomatology, such as nausea. One of the most interesting approaches to nausea adopted by traditional Chinese medicine is the stimulation of the acupoint PC6 *Neiguan.* Actually there are no studies in medical literature as to the efficacy of treating PC6 acupoint for gastrointestinal symptoms in migraine attacks. Our study aimed at verifying if pressure applied to the acupoint PC6 was effective on nausea during migraine. Forty female patients suffering from migraine without aura were enrolled, if nausea was always present as accompanying symptomatology of their migraine. The patients were treated randomly for a total of six migraine attacks: three with the application of a device, the Sea-Band^®^ wristband, which applies continual pressure to the PC6 acupoint (phase SB), and three without it (phase C). The intensities of nausea at the onset, at 30, 60, 120 and 240 min were evaluated on a scale from 0 to 10. The values were always significantly lower in phase SB than in phase C. Also the number of patients who reported at least a 50 % reduction in the nausea score was significantly higher in phase SB than in phase C at 30, 60 and 120 min. Moreover, the consistency of the treatment (response in at least two out of three treated attacks) was reached in 28 % patients at 60 min; in 40 % at 120 min and 59 % at 240 min. Our results encourage the application of PC6 acupressure for the treatment of migraine-associated nausea.

## Introduction

Migraine is a disabling neurological disorder considered by the World Health Organization as the 19th leading cause of all years lived with disability among both males and females of all ages, and as the 12th leading cause of years lived with disability among females of all ages [1]. Apart from pain, the disability caused by migraine is aggravated by accompanying symptomatology, such as gastro-intestinal symptoms, the most common being nausea and vomiting, to such an extent that their presence is one of the diagnostic criteria for migraine [2]. A telephone interview survey of 500 self-reported migraine sufferers found that nausea occurred in more than 90 % of all migraineurs; nearly one-third of these had nausea during every attack. Vomiting occurred in almost 70 % of all migraineurs; nearly one-third of these vomited in the majority of attacks. Indeed, 30.5 % of those who had nausea, reported that it interfered with their ability to take their oral migraine medication [3]. The American Study II stated that 73 % of the migraineurs studied reported to have suffered from nausea during attacks and that 29 % had vomited [4].

One of the most interesting approaches to nausea adopted by traditional Chinese medicine and, in particular, by acupuncture is the stimulation of the acupoint PC6 *Neiguan.* Indeed, there is documented evidence as to the efficacy of stimulating this point to alleviate chemotherapy-induced nausea and vomiting (CINV), postoperative nausea and vomiting (PONV) and motion sickness, both with acupuncture and acupressure. However, to the best of our knowledge, there are no studies in indexed medical literature as to the efficacy of treating PC6 acupoint for gastrointestinal symptoms in migraine attacks and particularly for nausea. Therefore, our preliminary study aimed at verifying if pressure applied to the point PC6 was effective on the presence of nausea during migraine attacks.

## Patients and methods

**Fig. 1:**
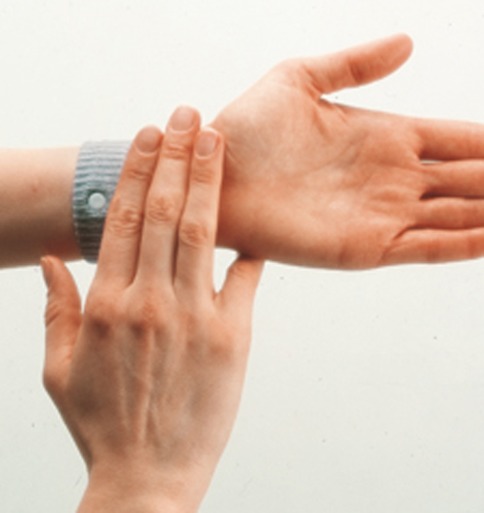
The localization of the point PC6 *Neiguan* and the correct positioning of the Sea-Bands^®^

A total of 40 female patients were enrolled into this study, after having given their informed consent, and all were suffering from migraine without aura, diagnosed according to the criteria established by the International Classification of Headache Disorders (ICHD-II) [2]. The patients were examined at the Women’s Headache Center, Department of Gynaecology and Obstetrics of Turin University. Inclusion criteria were: at least two migraine attacks per month for a 1-year period before enrollment; no more than 15 days of pain per month. The study had the maximum duration of 3 months. None of the patients were on prophylactic therapy, but were allowed to continue taking their usual symptomatic treatment. The patients’ medical history had to include the presence of nausea as accompanying symptomatology of their migraine, documented by a diary noting at least 1 month of attacks with nausea, prior to the inclusion in the study. Subjects taking antiemetics to control their nausea, whether as a single product or present as a compound in a combination product for the control of migraine, were excluded from the study. The patients enrolled were asked to fill in a dedicated diary recording the details of the length and intensity of the migraine attacks along with the accompanying symptomatology, paying particular attention to the presence of nausea. A device known as the Sea-Band^®^ was given to the patients to control their nausea. The Sea-Bands^®^ are elastic wristbands with a 1 cm protruding round plastic button; these devices apply continual pressure to the PC6 acupuncture point with the aim of decreasing, or completely eliminating nausea (Fig. 1). The PC6 point, also called *Neiguan*, is located on the anterior surface of the forearm, 3 fingers widths up from the first wrist crease and between the tendons of the flexor carpi radialis and palmaris longus. The Sea-Bands^®^ were applied bilaterally on both wrists on the *Neiguan* point, starting from the onset of the migraine attack and left in place for no less than 4 h, or for the whole attack period.

The patients were asked to document a total of six migraine attacks: three without the use of the Sea-Band^®^ wristbands (phase C, control) and three with the application of the Sea-Bands^®^ (phase SB). The sequence of the treatment given for the attacks (with, or without Sea-Bands^®^) was chosen at random according to a scheme provided by the computer and was applied to each single patient.

The section of the diary provided that covered the symptom of nausea was detailed to include information as to the time of symptom onset and symptom resolution, the intensity of nausea at the onset (T0), at 30 (T1), 60 (T2), 120 (T3) and 240 (T4) minutes evaluated on a scale from 0 to 10, where 0 indicated no nausea and 10 the maximum sensation of nausea.

Diary analysis was carried out by an impartial operator who did not know in which attacks the Sea-Bands^®^ were used or not. In this preliminary study, the analysis of diaries is focused only on nausea symptomatology.

The average values of nausea in phases C and SB were calculated at different times throughout the study and a statistical evaluation of the differences between the values obtained in T0, T1, T2, T3 and T4 in the two groups studied was performed using a non-parametric Friedman test for repeated measures.

Moreover, a non-parametric Wilcoxon test for paired data was always performed for each level of the variable ‘‘time’’ to evaluate the difference between phase C and phase SB. This test also took into consideration the average intensity of the three attacks in each of the two phases. All values given in the following text are reported as arithmetic means (±SEM). A Chi square test was applied for proportions. All analyses were performed using the Statistical Package for the Social Sciences (SPSS) software program.

## Results

Only 32 patients (mean age 39.65 years, range 19–61) completed the study. Four patients were lost to follow-up, three handed over a diary with incomplete, unreliable data and one patient did not suffer from any migraine attacks in the 3-month observation period. The Friedman test for repeated measures showed a highly statistically significant reduction in the intensity of nausea in the SB group (*p* < 0.001) during treatment (at T1, T2, T3 and T4).

**Fig. 2:**
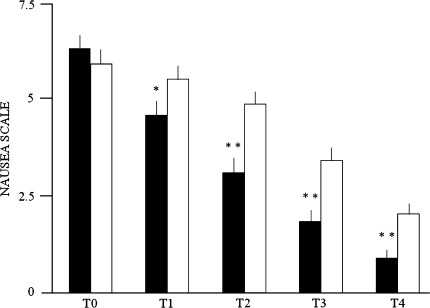
Average values of nausea score before treatment (T0), after 30 min (T1), after 60 min (T2), after 120 min (T3), after 240 min (T4), in phase SB (*black columns*) and in phase C (*white columns*). Non-parametric Wilcoxon test for paired data at T0, T1, T2, T3 and T4: at T0 *p* = 0.276, n.s.; at T1 *p = 0.006; at T2, T3 and T4 **p < 0.001

The Wilcoxon test for paired data showed that the nausea intensities were significantly higher in phase C than in phase SB (Fig. 2): after 30 min (T1 C 5.55 ± 0.36 vs. T1 SB 4.6 ± 0.40, *p* = 0.006), 60 min (T2 C 4.93 ± 0.33 vs. T2 SB 3.11 ± 0.40 *p* < 0.001), 120 min (T3 C 3.48 ± 0.35 vs. T3 SB 1.89 ± 0.31 *p* < 0.001) and 240 min (T4 C 2.05 ± 0.28 vs. T4 SB 0.93 ± 0.23 *p* < 0.001). There was no difference between groups at T0 (T0 C 5.96 ± 0.38 vs. T0 SB 6.36 ± 0.35; *p* = 0.276).

The number of patients who reported having had at least a 50 % reduction in the nausea score was: 0/32 at 30 min in phase C and 7/32 in phase SB (Chi square test: *p* = 0.16 RR 0.43; CI 95 % 0.32–0.58); 1/32 at 60 min in phase C and 15/32 in phase SB (Chi square test: *p* < 0.001 RR 0.37, CI 95 % 0.25–0.56); 11/32 at 120 min in phase C and 23/32 in phase SB (Chi square test: *p* = 0.003 RR 0.44, CI 95 % 0.24–0.80); 21/32 at 240 min in phase C and 27/32 in phase SB (Chi square test: *p* = 0.083 RR 0.55, CI 95 % 0.25–1.1). Moreover, when the consistency of the treatment (response in at least two out of three treated attacks) is taken into consideration, it was reached: in 9 patients (28 %) at 60 min; in 13 (40 %) at 120 min and in 19 (59 %) at 240 min. Noteworthy, the nausea was significantly reduced by acupressure in 3/3 attacks: in 5/32 patients (15 %) at 60 min; in 10/32 (31 %) at 120 min and in 17/32 (53 %) at 240 min.

## Discussion

Nausea is one of the most invalidating symptoms associated with migraine attacks. Some studies have reported that nausea was present in 73 to more than 90 % of the subjects studied and that almost one-third of these experienced nausea during every attack. Moreover, 30.5 % of the subjects who reported nausea indicated that its severity even interfered with their ability to take their oral migraine medication [3, 4].

Traditional Chinese medicine and especially acupuncture, stimulates some points that can be considered extremely valid from the point of view of nausea and/or vomiting control. In particular, the treatment of the acupoint PC6 *Neiguan* may be applied to this aim, even with the application of acupressure alone, as has been validated by various studies. International literature reports numerous studies on the efficacy of stimulating the acupuncture point PC6 and its capacity to reduce nausea under various clinical conditions. A Cochrane Review on PONV concluded by stating that, compared with sham treatment, acupoint stimulation significantly reduces nausea (RR 0.71, 95 % CI 0.61–0.83) and the need for rescue antiemetics (RR 0.69, 95 % CI 0.57–0.83) [5]. From a Cochrane Review on CINV, it emerged that acupressure is effective for both mean and worst acute nausea severity, and, therefore, acupressure is able to offer a no-cost, convenient, self-administered intervention for chemotherapy patients to reduce acute nausea [6].

On the basis of the data obtained in this study, the application of acupressure for the control of nausea during a migraine attack seems to be justified. Indeed, the application of the Sea-Bands^®^ on the acupoint PC6 Neiguan was observed to be effective in the control of nausea. The average nausea scores drop in the SB phase from 6.36 ± 0.35 in T0, to 4.60 ± 0.39 in T1, to 3.11 ± 0.40 in T2, to 1.88 ± 0.31 in T3 and to 0.92 ± 0.22 in T4. At each time step taken into consideration after the application of the Sea-Bands^®^, there was a statistically significant improvement over the non-treated phases. Moreover, there was a high percentage of responders to the treatment: i.e. 46.8 % at 60 min; 71.8 % at 120 min; 84.3 % at 240 min with a consistent response over time. Even when the fact that our study is both preliminary and open is taken into consideration, the results obtained seem to be encouraging and advocate the continuous application of PC6 acupressure in all migraine attacks with the accompanying symptom of nausea. Further controlled studies are, of course, required to validate the findings of this study.

## Abbreviations

CINV: Chemotherapy-induced nausea and vomiting
PONV: Postoperative nausea and vomiting
